# Compact Storage of Radioactive Cesium in Compressed Pellets of Zeolite Polymer Composite Fibers

**DOI:** 10.3390/ma11081347

**Published:** 2018-08-03

**Authors:** Masaru Ooshiro, Takaomi Kobayashi, Shuji Uchida

**Affiliations:** 1 Department of Materials Science and Technology, Nagaoka University of Technology, 1603-1 Kamitomioka, Nagaoka 940-2188, Japan; ohshiro@kasai-corporation.co.jp; 2 Kasai Co., Ltd., 578-3 Kawaguchi Akiha-ku, Niigata 956-0015, Japan; 3 Department of Chemistry and Biochemistry, National Institute of Technology, Fukushima College, Taira-kamiarakawa Nagao 30, Iwaki 970-8034, Japan; uchidas@fukushima-nct.ac.jp

**Keywords:** cesium adsorbed, radioactive cesium, safe storage, zeolite polymer composite fiber

## Abstract

To facilitate the safe storage of radioactive Cs, a zeolite–poly(ethersulfone) composite fiber was fabricated to be a compact storage form of radioactive Cs, and an immobilization was investigated with respect to the effects of volume reduction and stability of the fiber’s adsorbent matrix. Using compressed heat treatment at 100–800 °C for a zeolite polymer composite fiber (ZPCF) containing Cs, the fabrication changed its form from a fiber into a pellet, which decreased the matrix volume to be about one-sixth of its original volume. The Cs leakage behavior of the ZPCF matrix was examined in its compact pellet form for non-radioactive Cs and radioactive Cs when different fabrication conditions were carried out in the immobilization. The elution ratio of non-radioactive Cs from the matrix was minimal, at 0.05%, when the ZPCF was compressed with heat treatment at 300 °C. When using radioactive Cs for the compression at below 300 °C, the pellet form also had no elution of the pollutants from the matrix. When the compressed treatment was at 500 °C, the matrix exhibited elution of radioactive Cs to the outside, meaning that the plastic component was burning and decomposed in the pellet. A comparison of ZPCF and natural zeolite indicated that the compressed heating process for ZPCF was useful in a less-volume-immobilized form of the compact adsorbent for radioactive Cs storage.

## 1. Introduction

The severe accident that occurred on 11 March 2011 at the Fukushima Daiichi Nuclear Power Plant resulted from difficulties related to the catastrophic earthquake and the subsequent tsunami. The event damaged the plant, which released great amounts of radioactive ^134^Cs and ^137^Cs. Since then, the residual radioactivity has persisted as a hazard for local residents [1]. The huge volume of contaminants was estimated in 2013 as about 15−28 × 10^6^ m^3^ [2] and now in 2018 the radioactive Cs has been reduced in the outfields by the efforts of the decontamination process. It is known that the estimated amounts of radionuclides released into the atmosphere in 2012 in Fukushima were 6.1–62.5 PBq and 65–200 PBq for ^137^Cs and ^131^I, respectively. Especially, the Cs radioisotopes have been found frequently in aqueous radioactive wastes, mostly at levels exceeding the standards set for the areas. Because Cs belongs to a chemically similar group that includes sodium and potassium, ingestion of Cs radioisotopes can engender their deposition in tissues throughout the human body, thereby presenting an internal hazard to human health.

Over the years that have passed since the accident, external exposure to ^137^Cs, which has a long half-life of 30.5 years, has come to dominate radionuclide exposure. The trapped ^137^Cs wastes now present a health risk in Fukushima because the decontamination processes have emitted huge amounts of radioactive waste that remain in the environment. Along with increased concern related to the Cs radionuclide waste, people feel threatened in their life environments. Consequently, decontamination processes have continued to cope with the huge amounts of radioactive Cs. An effective mitigation method must be found through the consideration of attractive technologies. Among such methods, immobilization techniques have been presented for the remediation of radioactive Cs [3,4,5] (Awual et al., 2016; Kobayashi et al., 2016; Miah et al., 2010). For the large amounts of radioactive Cs that still exist, the development of some adsorption technology is needed in a safe form. Additionally, for the proper management and storage of radioactive waste, the volume reduction of secondary wastes has been especially important in the adsorbed wastes.

The methods for immobilizing radioactive Cs include solidification. In some studies of radioactive fly ash treatment, embedding radioactive materials in a solidified form was accomplished using a nanometallic Ca/CaO suspension for wastewater [6] (Reddy et al., 2014). Reportedly, pyrolytic carbon-coated zeolite is effective [7] (Stinton et al., 1983). Radioactive wastewater has been immobilized in a concrete matrix and in struvite ceramics [8] (Wagh et al., 2016). Moreover, immobilization has been achieved in a HZr_2_(PO_4_)_3_ matrix [9] (Nakayama et al., 2003), ash-based geopolymers [10] (Cozzi et al., 2013), and in rice husk silica geopolymers [11] (Lopez et al., 2014). Nevertheless, no report of the relevant literature has described immobilization by polymeric envelopment of a radioactive species with zeolite. Flammable wastes have been incinerated to accumulate enormous amounts of fly ash containing radioactive Cs. Our groups have reported that a zeolite polymer composite fiber (ZPCF) is an effective agent for treating water contaminated with radioactive Cs [12] (Ohshiro et al., 2017) and heavy metal ions [13] (Nakamoto et al., 2017) when the diluted pollutant was concentrated by the decontamination process in the outside of the field [4] (Kobayashi et al., 2016). However, post-adsorption in the ZPCF containing radioactive Cs presents storage problems for coming decades because a greater volume of wastes has to be stored. In post-adsorption processes for such radioactive Cs, reducing the volume becomes necessary and important for later storage processes. Therefore, several methods of immobilizing radioactive Cs in a matrix have been proposed as described above. For radioactive Cs remediation, the ZPCF used has been holding it strongly in the matrix even though the radioactive Cs was concentrated from an extra-diluted solution of radionuclides. Therefore, in the present study, the required compaction technology for ZPCF is described for the fiber’s volume reduction and its safe storage. The immobilization of ZPCF used for radioactive Cs was assessed in terms of the leakage of the pollutant from the matrix. Here, the tests were carried out by using an actual radioactive fly ash source. The results showed that the reduced-volume fiber has excellent capabilities for Cs immobilization.

## 2. Materials and Methods

### 2.1. Materials

**Figure 1:**
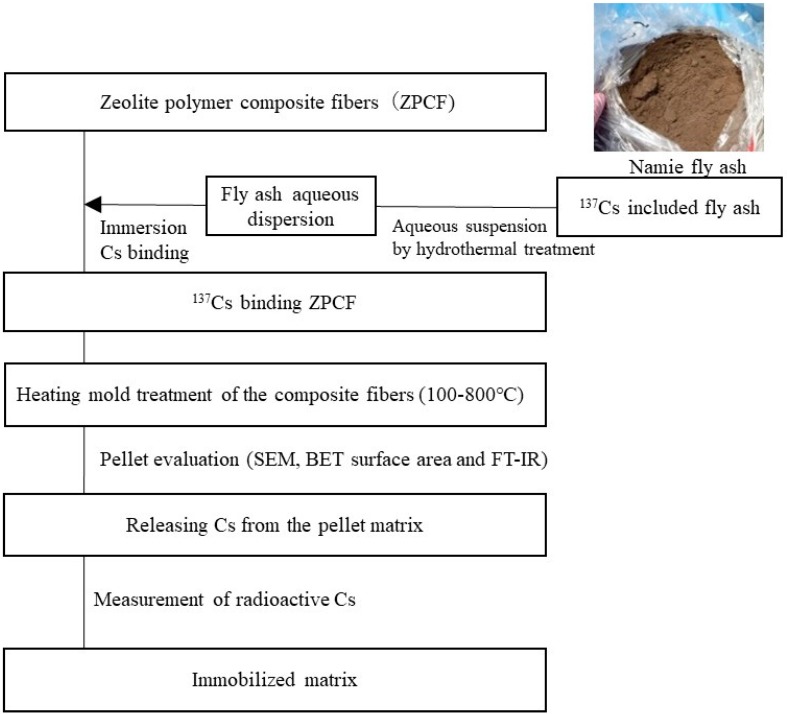
Flowchart of experiment procedure for Cs immobilization by heat treatment of zeolite polymer composite fibers and their Cs release processes. BET, Brunauer–Emmett–Teller.

Natural mordenite zeolite powder (≤100 μm) was purchased from Nitto Funka Trading Co. Ltd. (Miyagi, Japan). Poly(ethersulfone) (PES) was used as received (PES, MV = 50,000; BASF Japan Ltd., Ludwigshafen, Germany). *N*-methyl-2-pyrrolidone (NMP; Nacalai Tesque Inc., Kyoto, Japan) was used for the ZPCF without purification. The radioactive Cs adsorbed into the ZPCF was prepared as shown in Figure 1. Here, the ZPCF contained 59 wt% zeolite and 41 wt% was PES, which formed porous fibers. An aqueous solution containing radioactive Cs was prepared using hydrothermal extraction of fly ash for 2 h at 200 °C and 1.5 MPa. Here, the radioactive Cs fly ash was sampled in Namie, Fukushima (Figure 1). After the supernatant dispersed with fly ash was filtrated, the radioactive aqueous solution was used for experiments to fill the ZPCF. Then, the Cs-adsorbed fibers were prepared. The total radioactivity in the weighted fibers was measured in Becquerel per kilogram (Bq/kg) units. As Figure 1 shows, after Cs was bound to the ZPCF, a procedure for immobilization by compressed heat treatments of different temperatures was tested to create pellets. Then, Cs release was evaluated in addition to the matrix properties of the heat-treated matrix. The Cs adsorption process was conducted as follows by using radioactive fly ash (4 kg) having about 30,000 Bq/kg with Cs. The fly ash was dispersed in water (16 L) and filtered. Then, ZPCF (4 kg) was immersed in the Cs solution for 12 h. After this binding process, the ZPCF was used to measure the radioactive Cs concentration. For example, the radioactive Cs that remained in the fibers was 13,100–33,000 Bq/kg after the immersion process.

### 2.2. Characterization of Zeolite Polymer Composite Fibers

**Figure 2:**
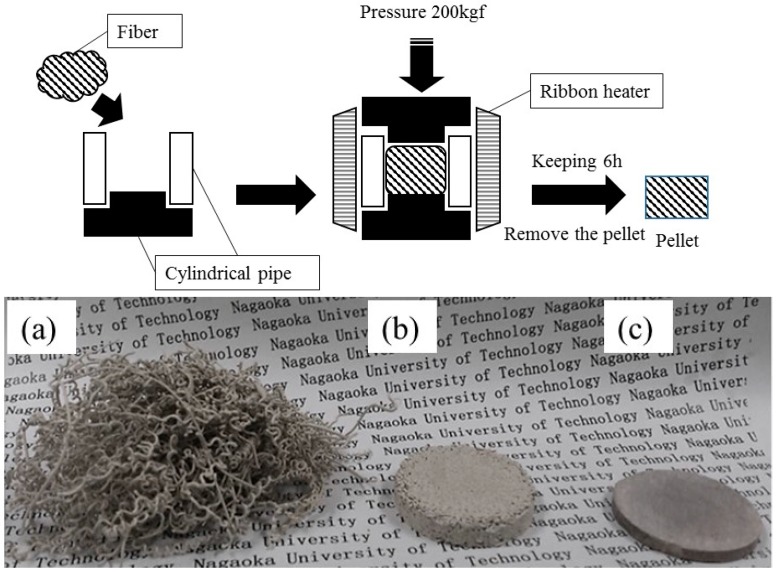
Schematic illustration of heating mold processes for preparation of the pellet matrix. The picture shows, respectively, fibers (**a**) before and (**b**,**c**) after heating mold processes at 100 °C and 300 °C [14].

To fabricate the ZPCF pellets enveloping the radioactive Cs, processes of immobilization and volume reduction were included in the heat molding process. The heat molding process is depicted in Figure 2. After the fibers (10 g) were pressed inside of a cylindrical stainless steel tube (50 mm height, 40 mm diameter, and 2.5 mm thickness), they were heated by a surrounding ribbon heater for 2 h at different temperatures of 100, 200, 300, 400, 600, and 800 °C. The temperature was measured using a thermocouple thermometer (FINE THERMO DG2N 100; HAKKO Ltd., Nagano, Japan). Then, the cover was pressed using a hydraulic press machine (P-16B Air Valve; Riken Seiki, Ojiya, Niigata, Japan) at 200 kg/cm^2^ for 6 h. After heat molding processing under pressure, the Brunauer–Emmett–Teller (BET) surface area and weight loss of adsorbents were measured. The pellet form was prepared using heat mold processing. For experimental procedures to assess radioactive Cs leakage from the fiber pellet matrix, a 10 g pellet matrix was immersed in 100 mL water at 20 °C. The fibers or the pellets, after accurate measurement of their weight, were washed 10 times with water and were immersed in 100 mL of water for 6 h and 24 h with stirring at 200 rpm. Then, the matrix and the solution were used to measure the resident Cs with a germanium semiconductor detector. The remaining and eluted Cs were estimated and then the value of the Cs eluted rate (%) was calculated using the following equation:

Cs eluted rate (%) = (*C* × *V*/*C*_0_ × *W*) × 100

where *C* is the Cs concentrations of eluted water, *V* denotes the eluted water volume, *C*_0_ represents the Cs concentrations of the fiber or pellet, and *W* is the fiber or pellet weight. Then, the released radioactive Cs was evaluated by measuring the Bq/kg amounts of the pellet and the washed water. The thermal analyses were carried out by Differential Scanning Calorimetry (DSC) (Thermo plus EVO2 DSC8231; Rigaku, Japan), Thermomechanical Analysis (TMA) (TMA-60; SHIMADZU, Kyoto, Japan), and Thermogravimeter-Differential Thermal Analysis (TG-DTA) (DTG-60; SHIMADZU, Kyoto, Japan). Here, the upper limitation of the elevated temperature in DSC was at 400 °C. The temperature of the TG-DTA was 480 °C in an aluminum pan. Unfortunately, setting DSC to 480 °C is impossible because at over 400 °C it damages the equipment with the aluminum pan.

In addition, scanning electron microscopy (SEM) images were taken (JSM-5310LVB; JEOL, Tokyo, Japan) of the ZPCF and the compacted pellet. Additionally, the sample was sputtered at 0.1 Torr for 40 s with Au for SEM measurements. A Fourier transform infrared spectrometer (FT-IR, IR Prestige-21 FTIR 8400s; Shimadzu Corp., Kyoto, Japan) was used with the KBr method. The N_2_ adsorption was analyzed using the Brunauer–Emmett–Teller (BET) surface area (Tristar II 3020; Micrometric Inc., Sarasota, FL, USA).

## 3. Results and Discussion

### 3.1. Immobilization of Radioactive Cesium by Heating Mold Processing of Composite Fibers

**Figure 3:**
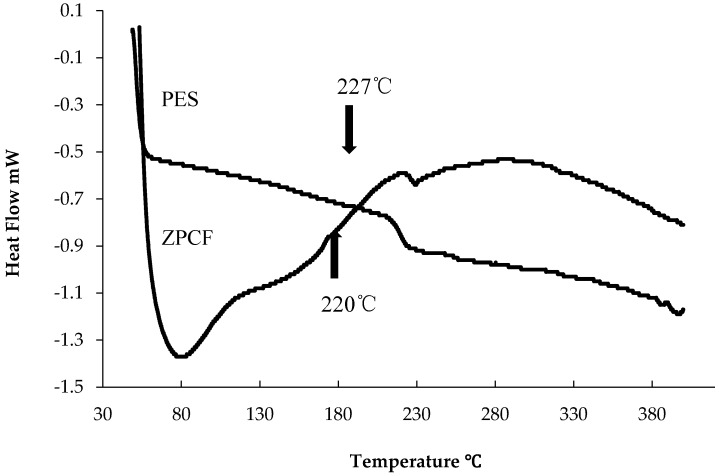
Results of Heat analysis of zeolite polymer composite fiber (ZPCF) and PES.

**Figure 4:**
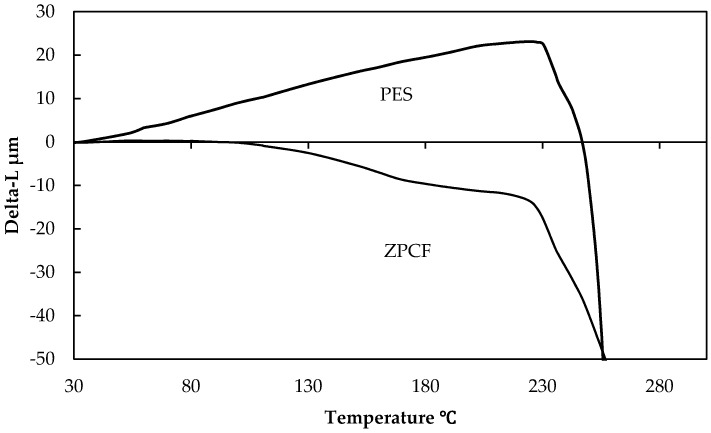
TMA measurement of PES and ZPCF at different temperatures.

As shown in Figure 2, for the schematic illustration of heating mold processes, the radioactive Cs was bound by the ZPCF in the dispersed fly ash solution. Then, the compacted pellet was shaped from the fibrous sample by heating it at different temperatures under pressure. The DSC curves of the PES fiber are shown in Figure 3 for the ZPCF and PES without zeolite. Endothermic peaks were found at 220 °C and 227 °C for the PES and the ZPCF in each DSC curve. This indicated that heating at over 230 °C led to the PES being melted. In the ZPCF, the observed temperature was found to be a little bit higher relative to that of the PES. It was noted that in the higher 220 °C and 227 °C range, the heat molding was reasonable to process and enabled the pellet form, while a pellet prepared at lower than 220 °C was only compressed by pressure. Additionally, it was apparent that the pellet form could be made to different densities at the 100 °C and 300 °C process temperatures. As shown in Figure 4, this was also supported by TMA measurement data. As a result of TMA measurement, the PES was found to be softened and melted from around 234–250 °C. Therefore, the TMA measurements were automatically stopped at over 250 °C. Here, the thermal DSC and TMA analyses could not obtain results at over 400 °C.

**Figure 5:**
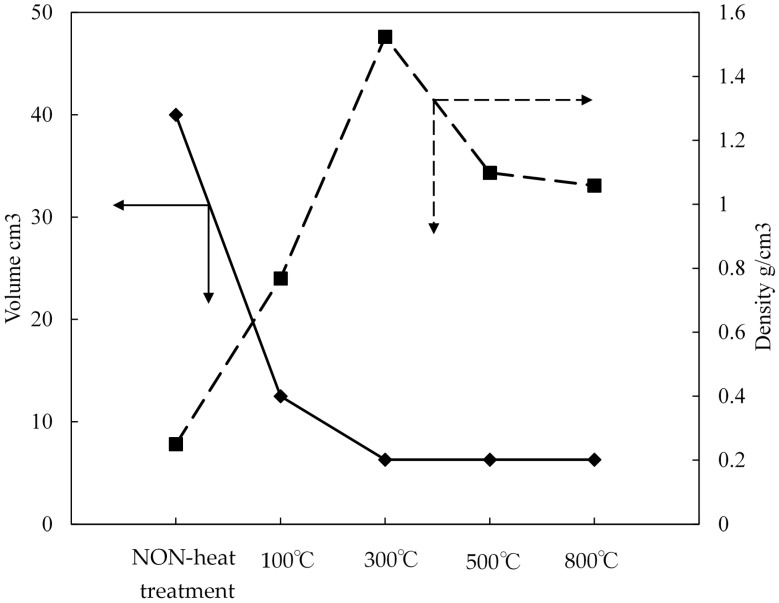
Relation between heating mold press temperature and the pellet volume and density.

Figure 5 presents the relation between the volume (cm^3^) and density (g/cm^3^) changes of the pellets. The ZPCF volume was decreased from 40 cm^3^ to 12.5 cm^3^ under the compressed heat molding process at 100 °C. Then, at over 300–800 °C, the volume changed by 6.3 cm^3^. The comparison indicated that the molding at 300 °C showed significant volume reduction to about one-sixth in the fabricated pellet. In addition, the pellet density increased from 0.25 to 1.52 g/cm^3^ after compression processing was implemented in the range of room temperature to 300 °C. This exactly was due to PES melting in the dense pellet formation as supported by Figure 3 and Figure 4. When the temperature was changed from 300 °C to 500 °C and 800 °C, the pellet density was decreased from 1.52 g/cm^3^ to 1.13 g/cm^3^ and then 1.1 g/cm^3^, respectively. Absolutely, the decrease was attributable to the polymer decomposition of the organic plastic PES. This was also supported in Figure 4 by the gradual decrease of the TMA results at over 300 °C in both curves.

**Figure 6:**
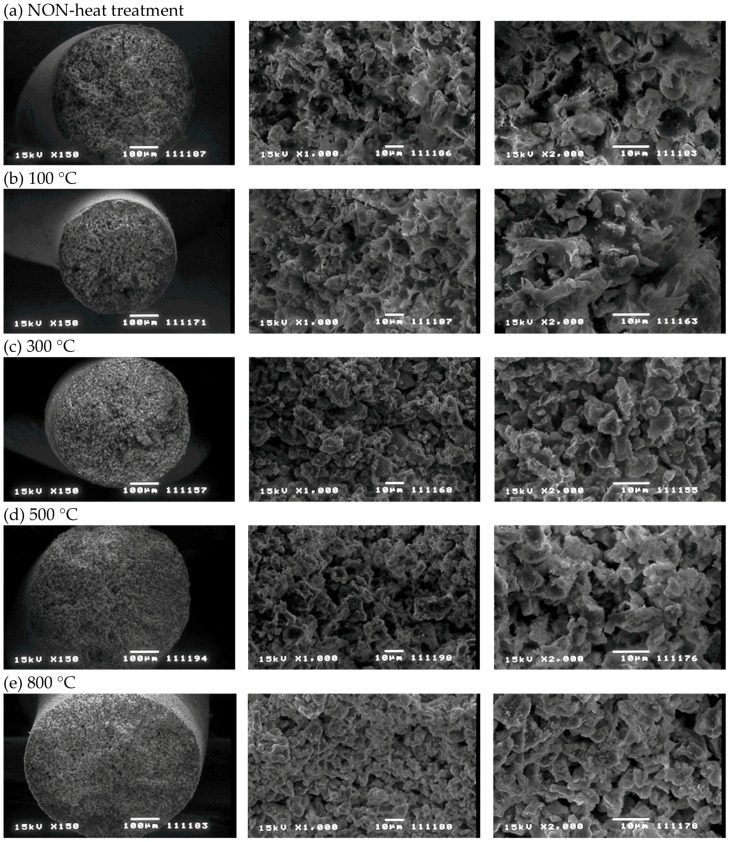
External view of pellets heated at different temperatures and corresponding SEM images [14]. (**a**) NON-heat treatment; (**b**) 100 °C; (**c**) 300 °C; (**d**) 500 °C and (**e**) 800 °C heat treatment.

**Figure 7:**
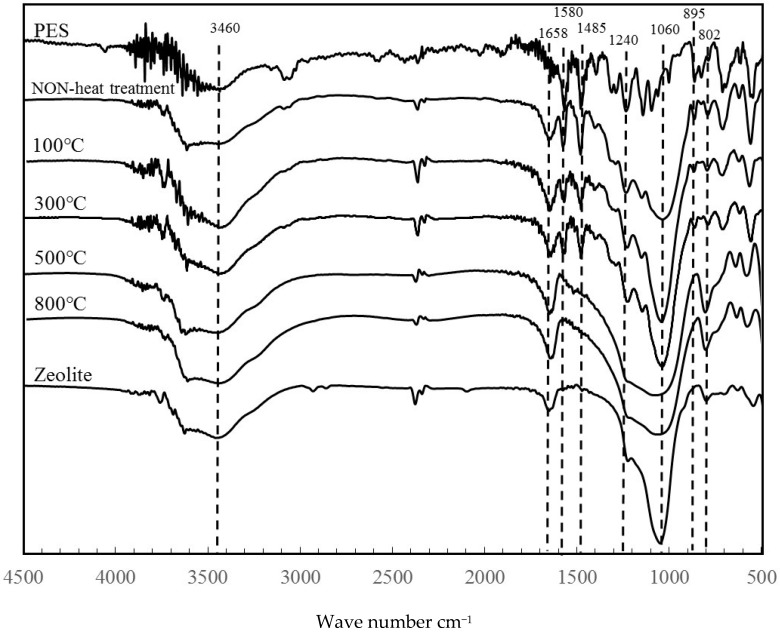
FT-IR spectra of ZPCF treated for 6 h at different temperatures.

**Figure 8:**
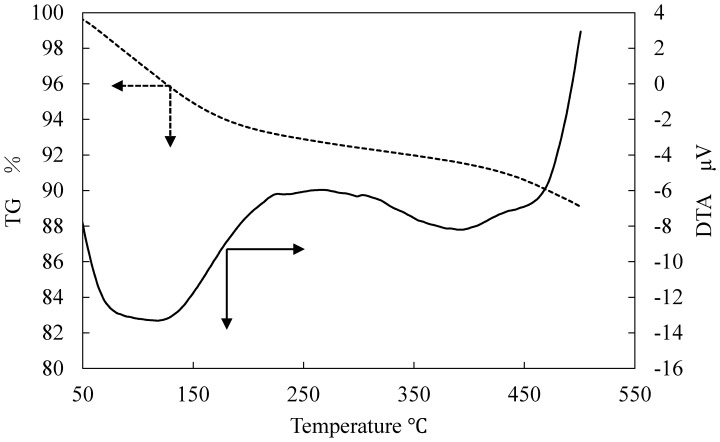
TG-DTA measurement of ZPCF at different temperatures.

Figure 6 portrays SEM images of the surface and cross sections of ZPCF treated at different temperatures for the molding processing. Low-temperature molding at 100 °C revealed that the zeolite powders were embedded in the PES medium as they were in the case of the non-heating mold. However, at temperatures higher than 300 °C, the images showed that the PES amounts decreased concomitantly with increasing temperature. At temperatures higher than 500 °C, the fibers were in a brittle state. Moreover, the polymer layer disappeared, reflecting the organic PES matrix’s decomposition. A cross-section view (Figure 6 left) of a pellet revealed the dense structure of the compressed fiber at 500 °C (d) and 800 °C (e). The FT-IR spectral patterns for the 800 °C sample (Figure 7) presented no peaks at 1580, 1485, and 895 cm^−1^ corresponding to PES in the ZPCF and peaks at 3460, 1658, and 1060 cm^−1^ corresponding to zeolite. Table 1 presents the respective assignments for their FT-IR peaks. Actually, band broadening was apparent in spectra obtained at 500 °C and 800 °C. Moreover, PES peaks were nonexistent. It was inferred from these results that the SiO_2_ component remained in the zeolite. The appearance of the 1658 cm^−1^ peak in the heated pellets might be assigned to water adsorbed from the atmosphere after heating. It is noteworthy that the appearance of the peaks at 1580 and 1485 cm^−1^ is temperature-dependent. At 800 °C, both PES peaks disappeared, meaning that the PES in the pellet was burned out of the material. Therefore, the spectra retained broad peaks at 1060 cm^−1^ and at 802 cm^−1^ for the Si–O–Si and Al–O bands of zeolite, respectively. As seen in Figure 8 for the TG-DTA measurement of ZPCF in the range of 50–500 °C, an endothermic peak and depletion of TG considered to be dehydrated from zeolite were observed up to 250 °C. After 450 °C, the exothermic peak and a decline of TG can be confirmed in the 350–500 °C range. This was presumed to be caused by the combustion of PES.

**Figure 9:**
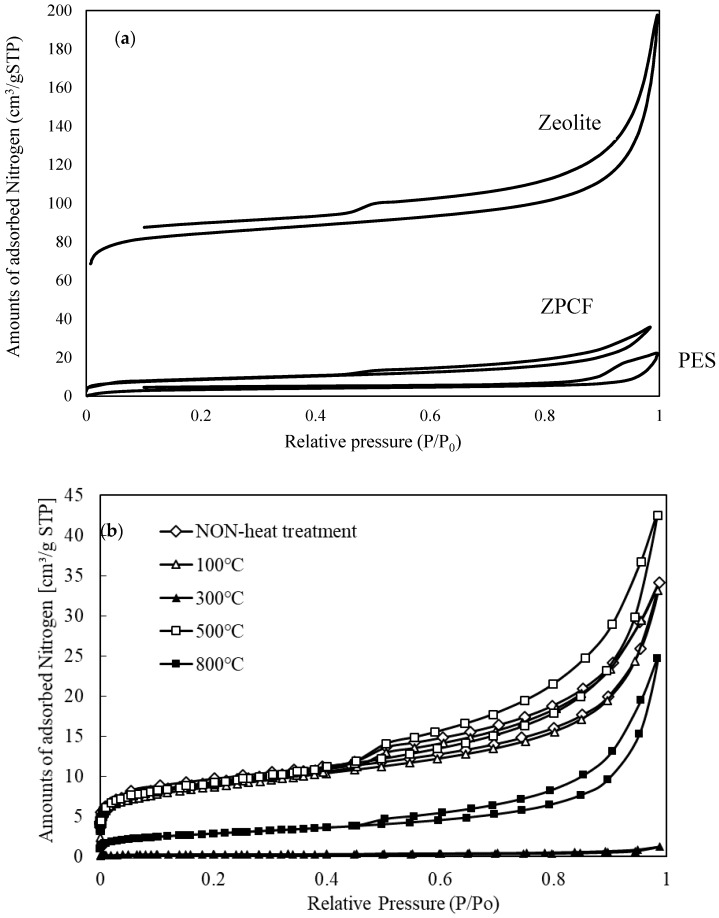
N_2_ adsorption isotherms of (**a**) PES and ZPCF and (**b**) pellets obtained with heat mold treatment at different temperatures.

To evaluate the porous and dense properties of the fibers and pellets, nitrogen (N_2_) adsorption and desorption were measured at different pressures. Figure 9 portrays the N_2_ adsorption isotherm of the PES and ZPCF in the absence (a) and presence (b) of heat molding processing. According to the isotherms, their samples presented that the adsorption behavior followed that of the type 2 isotherm, reflecting the presence of a macroporous structure [5] (Kobayashi et al., 2016). Compared to the PES and zeolite shown in Figure 9a, it was apparent that the PES fiber had a lower capacity for N_2_ adsorption amounts relative to the zeolite powder. As Figure 9b shows, the pellets molded at 500 °C and 800 °C showed similar isotherm curves to that of zeolite, as was true also for the non-heat treatment. In the different pellet samples at 500 °C and 800 °C, the amounts of adsorption of N_2_ were changed, although the shapes of the isotherms were almost similar. However, it was seen that the values of the pellet at 800 °C were lower than those at 500 °C. This strongly suggested that the numbers of the mesopores in the pellet at 800 °C were much lower than the numbers of mesopores in the pellet at 500 °C. This meant that the 800 °C molding destroyed the porous structure of the pellet. However, it is noteworthy that the curve of the pellet heated at 300 °C had lower amounts of N_2_ adsorption. Consequently, the melted PES penetrated and covered the zeolite pores causing the zeolite volume to decrease. As seen in Figure 4 and Figure 8, this was strongly suggested by the TMA and DTA results. Also, the FT-IR results of different temperatures showed decomposition of the PES components at temperatures over 500 °C. It is noteworthy that the N_2_ adsorption amounts were somewhat higher at the temperatures of 500 °C and 800 °C, which indicates that the PES component in the pellets decomposed gradually because the temperature was higher than around 300 °C and that it increased concomitantly with increasing temperature to 800 °C. Therefore, the PES component decomposition occurred gradually with increasing temperature. The results obtained at 800 °C showed that the zeolite surface was exposed without envelopment by the PES layer.

Table 2 presents the BET surface area and weight reduction of the ZPCF pellets heated at different temperatures. The BET surface area of the ZPCF was 32 m^2^/g before heat molding processing. The value was remarkably lower at 300 °C because the melted PES enveloped the porous structure of the zeolite. The pellet structure became denser. At 500 °C, however, the surface area value increased to 32 m^2^/g, suggesting that little zeolite remained after heating. Furthermore, since the heating treatment decomposed the PES component and thereby exposed the zeolite surface, the experimental results of the ZPCF weight reduction at 500 °C and 800 °C were, respectively, 30.8% and 33.3% after heat molding processing. This was due to the polymer being well-decomposed at over 500 °C. However, the value of weight reduction at 300 °C was 2.17 wt %, meaning that the PES had decomposed only a little. Figure 4 depicts the TMA results, which show that the PES of the thermoplastic polymer was melted at temperatures higher than 300 °C. Then, the melting PES seemed to cover the zeolite powder. Therefore, from the weight reduction results, one can reasonably infer that the melted PES surrounding the zeolite powders produced an enveloping layer that decreased the zeolite surface area. Table 2 contains the adsorbent concentration the radioactive Cs of the pellet matrix. At 500 °C and 800 °C, the values of the radioactive Cs were 21,200 and 22,400 Bq/kg. The results of radioactive Cs concentration were higher at 500 °C and 800 °C than those at 100–300 °C. This was due to weight reduction by the PES decomposition.

### 3.2. Immobilization of Radioactive Cesium in ZPCF Pellets

Table 3 presents values of the radioactive Cs amounts observed with and without heat molding processing. The ZPCF adsorbed up to 14,000 Bq/kg of Cs. The Cs concentration amounts were increased as the temperature increased after heat molding processing. It was seen that the increment was observed between 300 °C and 500 °C for radioactive Cs concentration. The increased concentration derived from the fact that the PES weight was lower at higher temperatures. Also, the weight reduction between 300 °C and 500 °C was remarkable. Table 3 contains values for the eluted concentration of radioactive Cs from the pellets when they were washed in distilled water. The values of the eluted Cs in water phase were measured. It was noted that the values of the radioactive Cs concentration from the pellet were less than 1 Bq/L at 100 °C and 300 °C. In contrast, the values were 60 and 62 Bq/L for the washed pellet molded at 500 °C. This result strongly indicated that adsorbed radioactive Cs was released from the matrix to the water.

**Figure 10:**
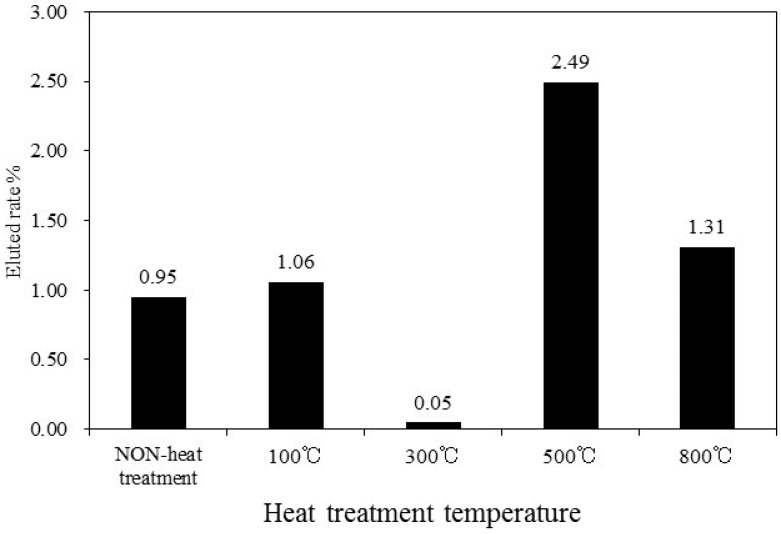
Elution rates of non-radioactive Cs from heat molding treatment of ZPCF [14].

The ZPCF’s form can be changed to a pellet by heat molding processing. Therefore, it was interesting to observe the radioactive Cs immobilization’s efficiency in the matrix. To measure changes in the radioactive Cs concentration, Cs release tests were conducted in water for each sample. Figure 10 shows the elution rate (%) of non-radioactive Cs. After the pellet was washed with diluted water for 6 h, the elution rate (%) was evaluated. Furthermore, Table 3 shows the rate of radioactive Cs elution from pellet matrixes molded at different temperatures. These results indicated that elution of the Cs from the fibers and pellets depends strongly upon the heat molding process temperature. The rate of elution of the Cs was extremely low, about 0.05%, at 300 °C relative to 0.95% at 100 °C and 2.49% at 500 °C. Therefore, it was apparent that the melted PES influenced the Cs release. These results exhibited the same tendency as that for results of the surface area as presented in Table 2. Therefore, for the 300 °C treatment, envelopment within the polymer layer was effective for Cs immobilization in the pellet matrix. At 800 °C, because heat processing greatly decomposed the PES layer surrounding the zeolite powders, a compacted pellet might be effective to fix the Cs component with the zeolite powder.

**Figure 11:**
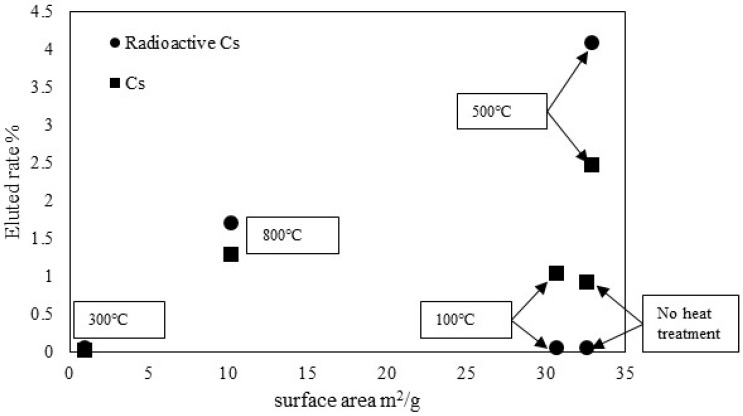
Relationship between elution rates of radioactive Cs and non-radioactive Cs and surface area from different heat-molding temperatures of ZPCF.

Table 3 presents results of radioactive Cs release tests conducted for 6 h and 24 h using different Becquerel per kilogram amounts of ZPCF. The table shows radioactive amounts as evaluated at 0 h and at different times before and after water washing. The elution rates (%) are values obtained at 24 h. The elution rates were similar to those found for non-radioactive Cs. For example, the ZPCF had 14,200 Bq/kg before washing for the non-heat treatment sample. The sample was 14,000 Bq/kg at 6 h and 24 h after washing in water. For the pellet sample prepared at 300 °C, the value of 15,300 Bq/kg after washing represented almost no change from the original 15,500 Bq/kg. However, after mold treatment at 500 °C, the value of 21,200 Bq/kg was increased to 29,800 Bq/kg at 6 h and 24 h. Table 3 shows that the observed weight reduction was about 30% after the elution tests for 500 °C and 800 °C, demonstrating that the component remaining after burning the PES from the pellet was a soluble substance, such as calcium salt. The radioactive Cs concentration was increased in the pellet. Furthermore, the case at 800 °C showed a similar phenomenon. Figure 11 summarizes the relationship between the rate of Cs release and the surface area m^2^/g. Apparently, the lower elution was due to the lesser surface area. When the molding temperature was increased from 300 to 500 °C, the surface area was also increased. The increase was due to decomposition of the PES and then exposure to the porous zeolite site. This meant that the high porosity of the PES plastic shape had kept the elution of Cs and decomposition much lower from the heat molding system on the experimental time scale. At 300 °C, the reason for the lower density was the melting of the PES that covered the porous zeolite site, meaning that a covering effect decreases Cs elution. In the case of the 100 °C molding treatment, the PES was not melted, keeping a higher surface area. Thus, the leaking of the radioactive Cs, which was equivalently the same level as that at 300 °C, becomes of a lower concentration of the immobilization, at about 20,000 Bq/kg. However, that of the non-radioactive Cs concentration was at 86.1 g/kg, meaning that it was extremely higher than that of radioactive Cs. Therefore, the leaking Cs occurred at 1.06%.

In conclusion, heat molding processing of ZPCF containing Cs efficiently immobilized radioactive Cs in pellets formed at temperatures lower than 300 °C. However, at temperatures higher than 300 °C, the immobilization effect was less pronounced. Furthermore, the results showed that the heat molding processing reduced the pellet volume. The results presented herein suggest that the ZPCF was a better matrix material for fixing radioactive Cs.

## 4. Conclusions

This study examined the immobilization of radioactive Cs by heated mold treatment of ZPCF for safe storage. The properties of pellets formed at different temperatures using heat molding processing were compared. An effective decrease of the eluted Cs from the pellet was observed at 300 °C, suggesting that the melted PES at 300 °C enveloped the zeolite, thereby fixing the radioactive Cs. This inference was supported by several observations of the ZPCF pellet’s surface area, which decreased because of envelopment by the melted polymer surrounding the zeolite powders. Actually, elution of the radioactive Cs was extremely low: less than 0.07–0.08%. Moreover, a volume reduction to 1/6 was achieved by heat molding of the ZPCF.

## Figures and Tables

**Table 1:** Infrared adsorption peaks of zeolite and PES.

Wavenumber (cm^−1^)	Component in ZPCF	Assignment
3460	Zeolite	OH st.
1658	zelite	Si–O–Si st.
1580	PES	C=O st.
1485	PES	C–O st.
1240	PES	SO st.
1060	zeolite	Si–O st.
895	PES	Polymer group C–C st.
802	zeolite	Al–O st.

st.: stands for stretching.

**Table 2:** Relation between the BET surface area, weight reduction, and radioactive Cs concentration of zeolite polymer composite fibers [14].

Heat Treatment Temperature	BET Surface Area m^2^/g	Weight Reduction %	Adsorbent Concentration Bq/kg	
			Before	After
NON-heat treatment	32.5	0	14,000	14,200
100 °C	30.6	0.67	14,000	13,100
300 °C	0.9	2.17	14,000	15,300
500 °C	32.8	30.8	14,000	21,200
800 °C	10.1	33.3	14,000	22,400

**Table 3:** Elution rate of radioactive Cs from heat treatment of ZPCF [14].

Sample	Adsorbents Concentration of ZPCF Bq/kg (Weight Reduction Rate %)			Eluted Solution Concentration of Water Bq/L		Elution Rate %
	0 h	6 h	24 h	6 h	24 h	
NON-heat treatment	14,200	14,000 (0.1%)	14,000 (0.1%)	<1	<1	0.07
100 °C	13,100	13,300 (0.0%)	13,300 (0.1%)	<1	<1	0.08
300 °C	15,300	15,500 (0.0%)	15,500 (0.1%)	<1	<1	0.07
500 °C	21,200	29,800 (29.1%)	29,800 (29.2%)	60	62	4.10
800 °C	22,400	33,000 (39.8%)	33,000 (30.0%)	25	26	1.73
zeolite	24,500	22,000 (0.1%)	22,000 (0.0%)	274	280	11.42
